# Deep Transfer Network with Multi-Space Dynamic Distribution Adaptation for Bearing Fault Diagnosis

**DOI:** 10.3390/e24081122

**Published:** 2022-08-15

**Authors:** Xiaorong Zheng, Zhaojian Gu, Caiming Liu, Jiahao Jiang, Zhiwei He, Mingyu Gao

**Affiliations:** 1 School of Electronic Information, Hangzhou Dianzi University, Hangzhou 310018, China; 2 Zhejiang Provincial Key Lab of Equipment Electronics, Hangzhou 310018, China

**Keywords:** fault diagnosis, rolling bearing, transfer leaning, domain adaptation

## Abstract

Domain adaptation-based bearing fault diagnosis methods have recently received high attention. However, the extracted features in these methods fail to adequately represent fault information due to the versatility of the work scenario. Moreover, most existing adaptive methods attempt to align the feature space of domains by calculating the sum of marginal distribution distance and conditional distribution distance, without considering variable cross-domain diagnostic scenarios that provide significant cues for fault diagnosis. To address the above problems, we propose a deep convolutional multi-space dynamic distribution adaptation (DCMSDA) model, which consists of two core components: two feature extraction modules and a dynamic distribution adaptation module. Technically, a multi-space structure is proposed in the feature extraction module to fully extract fault features of the marginal distribution and conditional distribution. In addition, the dynamic distribution adaptation module utilizes different metrics to capture distribution discrepancies, as well as an adaptive coefficient to dynamically measure the alignment proportion in complex cross-domain scenarios. This study compares our method with other advanced methods, in detail. The experimental results show that the proposed method has excellent diagnosis performance and generalization performance. Furthermore, the results further demonstrate the effectiveness of each transfer module proposed in our model.

## 1. Introduction

Bearing is an essential component of rotating machinery and plays a key role in industry. Due to the complex working environment and endless work, bearing failures cannot be avoided. Therefore, in order to ensure normal industrial production, it is important to accurately diagnose bearing faults. Deep neural network based methods have shown excellent performance in fault diagnosis tasks for rotating machinery [1,2,3] and other fields [4] due to their powerful feature capture capabilities. According to [5], the networks used in bearing fault diagnosis can be roughly divided into convolutional neural network (CNN) [6], auto encoder (AE) [7] and deep belief network (DBN) [8]. Although the positive results have been achieved, they still suffer from a problem in real-world scenarios, i.e., the domain shift. Specifically, the distribution of bearing training data and test data collected from variable operating conditions differs significantly. As a result, when a model trained on the training data is deployed to test data from different working conditions, the performance of the deep learning model is greatly limited. In other words, the feature space inconsistency between bearing training data and test data causes the trained model to fail to generalize to the test set.

To counter this domain shift issue, bearing fault diagnosis based on domain adaptation (DA) [5] offers a workable solution, which learns the common fault features of the source and target domain data in different working scenarios. Since the source and target domains have the same fault type in this method, it is possible to learn the classification boundaries of the target domain feature space based on the source domain and the fault classifier, which can successfully separate these domain-invariant features according to category boundaries. Due to the inherent similarity of fault features under different working conditions, the shared features present in both domains allow for some feasibility of domain adaptation [9]. Othman et al. [10] proposed a deep transfer learning model, in which a three-layer autoencoder was used to extract common fault features of the source and target domains for bearing fault identification, with a maximum mean discrepancy (MMD) metric as the main optimization objective. By optimizing the module, the feature space was aligned and the fault classifier utilized the information contained in the domain-invariant features to successfully implement fault diagnosis. An et al. [11] proposed a one-dimensional convolutional neural network to guide common fault representations. To improve network generalizability, the correlated alignment loss (CORAL) was considered as a regularization item to solve the domain shift problem due to inconsistent working conditions. Additionally, to simulate real production conditions, they added Gaussian noise to the input vibration signal. Even when disturbed by the noise, their model still showed superior fault-discriminative performance. Zhu et al. [12] added two additional layers to the CNN to extract task-specific features. By combining multiple Gaussian kernels, the training loss was calculated more accurately. In practice, they deployed different optimization objectives to train the model, also improving accuracy in bearing fault diagnosis. Wan et al. [13] proposed an improved deep network as the feature extractor to remove industrial noise from bearing signals and extract the a priori knowledge. Moreover, they constructed corresponding domain discriminators according to each type of fault. By adversarial training of the domain discriminator and the feature extractor, a more adequate alignment of the feature space was achieved.

Alignment of the feature space is the key to these domain adaptive approaches. As a sub-topic of domain adaptation, joint distribution adaptation (JDA) has been proposed for more levels of feature space alignment. It considers both global feature space alignment and fault category-specific feature space alignment to adapt the fault classifier to changing working conditions. Benefiting from this design, JDA methods perform with high accuracy [13]. However, there are still some problems that need to be addressed, as follows.

1. Most feature representations in JDA methods fail to adequately contain information about the fault types. They often ignore some fine-grained information, such as the size and location of the fault, etc. Although some methods take into account fine-grained information, most usually face problems of slow and difficult convergence [14,15], because the adversarial training process is complicated with two stages;
2. Since the joint distribution cannot be directly measured, most JDA methods calculate the sum of the marginal distribution distance and the conditional distribution distance, which is approximate to the joint distribution distance. Due to the approximation, accurate feature representation is critical in cross-domain scenarios. On one hand, when the bearing signals are collected from widely varying working conditions, the overall feature representations that represent marginal distribution are more important. On the other hand, when from similar conditions, feature representations of conditional distribution specific to each fault class are more critical. However, existing JDA methods have rarely investigated feature representation under various working conditions.

**Figure 1:**
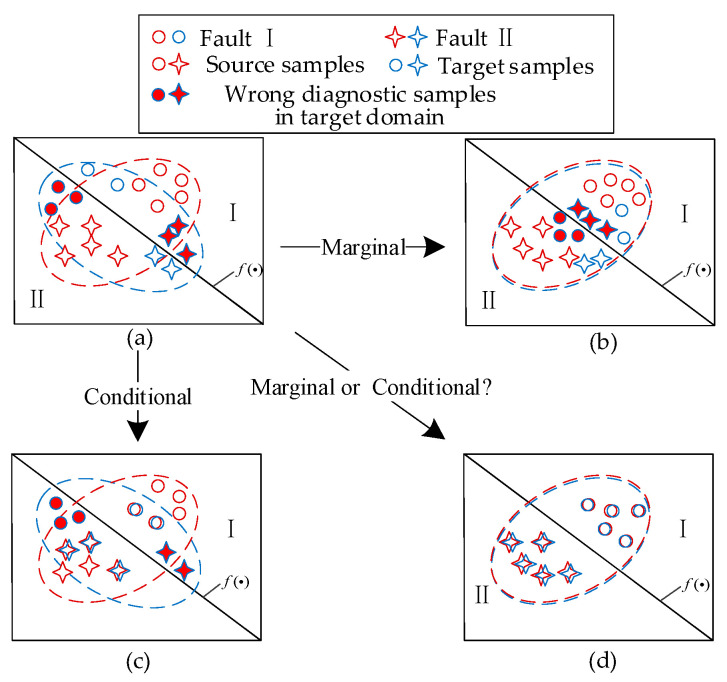
Intelligent fault diagnosis: (**a**) without dynamic distribution adaptation; (**b**) with marginal distribution adaptation; (**c**) with conditional distribution adaptation; and (**d**) with dynamic distribution adaptation.

To address these issues, a deep convolutional multi-space dynamic distribution adaptation (DCMSDA) model is proposed for bearing fault diagnosis. To adequately align the sample distribution, we propose a detailed feature extraction scheme and two optimization metrics. Figure 1b shows that marginal distribution adaptation aligns the global feature representations between domains, while Figure 1c shows that conditional distribution adaptation focuses on the category-specific feature representations. Additionally, Figure 1d shows that our model adequately aligns feature representations by automatically selecting the appropriate adaptation method, when faced with an unknown target domain from various working conditions. It can be clearly seen that the inadequate feature alignment problems in JDA methods can be effectively solved, which further enables the domain shift problem to be alleviated. The main contributions of this paper are as follows:

1. A marginal feature extraction module and a multi-space conditional feature extraction module are proposed to guide powerful feature representation of fault information. Based on these modules, the mapping effect of features at multiple scales is achieved;
2. A multi-kernel maximum mean discrepancy (MK-MMD) and a local maximum mean discrepancy (LMMD) are introduced as metrics to adjust the marginal distribution and the conditional distribution, respectively. By optimizing the objectives together, the distribution discrepancies within the extracted features can be reduced;
3. An adaptive coefficient is designed to dynamically measure the alignment proportion of feature representations. It reweights the fault feature representations by the construction of two domain discriminators, improving the generalization performance in complex cross-domain scenarios.

The details of the proposed model and its optimization objective are introduced in Section 2. The comparative experiments and ablation experiments are presented in Section 3. Finally, the conclusions and future work are provided in Section 4.

## 2. Research Methods

### 2.1. Proposed Framework

**Figure 2:**
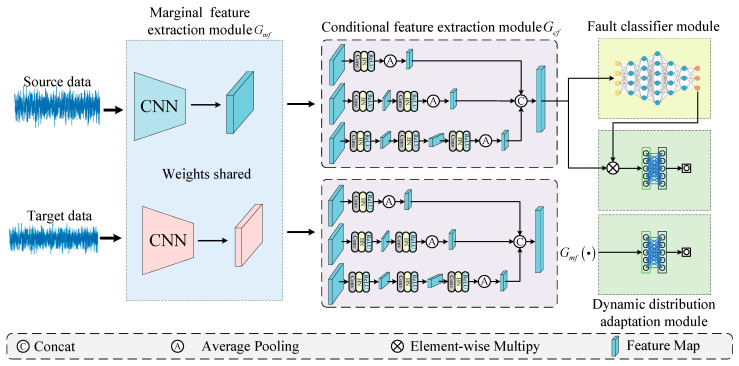
Framework of the proposed model.

The structure of our DCMSDA model is shown in Figure 2. The deep convolutional multi-space dynamic distribution adaptation network consists of four parts, including the marginal feature extraction module, the conditional feature extraction module, the dynamic distribution adaptation module and the fault classification module. The two feature extraction modules are composed of multiple stacked convolutional layers; the fault classification module is a fully connected layer with Softmax activation function; and the dynamic distribution adaptation module contains two domain discriminators, which consist of three fully connected layers with Sigmoid activation function. Given the raw time–domain signal as input, the marginal and conditional feature extraction modules are used to extract the features of the marginal and conditional distributions, respectively. After that, the fault classification module classifies the extracted fault features and outputs the classification vectors. During the training process, the proposed dynamic distribution adaptation module uses two metrics to measure the distribution discrepancies of domains. Moreover, to calculate the adaptive coefficient, which dynamically guides the feature extraction modules to extract more domain-invariant features, two domain discriminators are constructed to determine the domain to which the above features belong. The primary transfer modules introduced in the model include the feature extraction modules and the dynamic distribution adaptation module, which are described in this Section 2.1. The training process of the model is described in detail in Section 2.2.

#### 2.1.1. Feature Extraction Module

Deep neural network layers make the extracted features more task-specific dependent, while shallow layers learn general features [16,17]. To fully extract feature information, the marginal feature extraction module and the conditional feature extraction module were designed. The features extracted by the marginal feature extraction module were further fed into the conditional feature extraction module. In detail, the marginal feature extraction module is a shallow convolutional neural network, so the extracted features contain more general information with apparent marginal distribution discrepancies, which is called marginal features in this paper. The conditional feature extraction module was built by a multi-space convolutional neural network, which fuses features at different scales to produce conditional features with rich semantic and detailed information. Therefore, conditional features have significant differences to marginal features, enhancing the feature representation in fault classification tasks.

**Figure 3:**
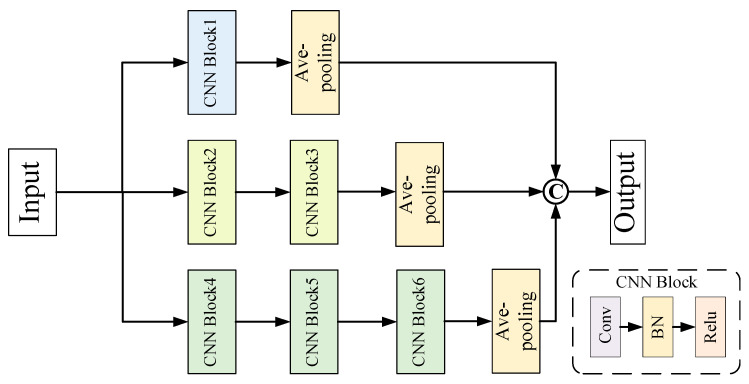
Structure of the conditional feature extraction module.

The marginal feature extraction module adopts ResNet-18 1D [5] to extract fault features. The conditional feature extraction module consists of three conditional feature extractors on three spaces, as shown in Figure 3. Here, multiple small convolutional layers with different levels are stacked. To present the model structure more clearly, Table 1 shows details of the implemented conditional feature extraction module. Since the three conditional feature extractors have different depths, convolutional kernel sizes and parameters, richer feature representations with semantic and detailed information can be extracted for the fault diagnosis task. The conditional features learned in the three spaces are fused together to form high-level features, providing informative cues for the fault classifier.

#### 2.1.2. Dynamic Distribution Adaptation Module

The proposed dynamic distribution adaptation module has three interesting elements: MK-MMD focuses on the global distribution discrepancy [18,19] and is suitable for the alignment of the marginal feature distribution; while LMMD focuses on the relationship between two sub-domains within the same category [14], and is appropriate for the conditional feature distribution. In addition, an adaptative coefficient is deployed to intuitively reweight the marginal distribution alignment and the conditional distribution alignment.

MK-MMD is used to optimize the marginal feature distribution, which can be formulated as follows:

(1) $$L_{m}=d_{k}^{2}\left(P\left(x_{s}\right),P\left(x_{t}\right)\right)≜{\left\|E_{P\left(x_{s}\right)}\left(\phi \left(G_{mf}\left(x_{{}^{i}}^{s}\right)\right)\right)-E_{P\left(x_{t}\right)}\left(\phi \left(G_{mf}\left(x_{{}^{i}}^{t}\right)\right)\right)\right\|}_{{ℋ}_{k}}^{2}$$

where $x_{i}^{s}$ and $x_{i}^{t}$ represent the i-th source-domain sample and the i-th target-domain sample, respectively, which obey the marginal probability distribution $P\left(x_{s}\right)$ and $P\left(x_{t}\right)$. $G_{mf}\left(\cdot \right)$ represents the feature representation extracted by marginal feature extractor, ${ℋ}_{k}$ represents reproducing kernel Hilbert space (RKHS), ϕ(·) represents the feature mapping of original samples to RKHS, and E represents the mathematical expectation of the two datasets in RKHS.

LMMD is applied to measure the conditional distribution distance on three spaces, which is expressed as follows:

(2) $$L_{c}=d_{k}^{2}\left(Q\left(x_{s}^{}\right),Q\left(x_{t}^{}\right)\right)≜\frac{1}{C}{\sum_{c=1}^{C}\left\|\sum_{x_{{}^{i}}^{s}\in D_{s}}\left(\omega_{i}^{s,c}\phi \left(G_{cf}\left(x_{{}^{i}}^{s}\right)\right)\right)-\sum_{x_{{}^{i}}^{t}\in D_{t}}\left(\omega_{i}^{t,c}\phi \left(G_{cf}\left(x_{{}^{i}}^{t}\right)\right)\right)\right\|}_{{ℋ}_{k}}^{2}$$

where $Q\left(x_{s}\right)$ and $Q\left(x_{t}\right)$ denote conditional probability distribution, $G_{cf}\left(\cdot \right)$ represents the feature representation extracted by conditional feature extractor, $\omega_{i}^{s,c}$ and $\omega_{i}^{t,c}$ represent the weight of the c-th category data $x_{{}^{i}}^{s}$ and $x_{{}^{i}}^{t}$, respectively. The source domain data use the real labels to calculate the corresponding weights, and the target domain data use the pseudo-labels output by the fault classifier to calculate weights.

The two domain binary discriminators classify the domain to which marginal features and conditional features belong. Here, we refer to them as the marginal domain discriminator and the conditional domain discriminator. The optimization objective of the the marginal domain discriminator is calculated by:

(3) $$\begin{array}{l} L_{md}=\left(G_{md}\left(G_{mf}\left(x_{{}^{i}}^{}\right)\right),d_{i}\right)=-d_{i}\log\left(G_{md}\left(G_{mf}\left(x_{{}^{i}}^{}\right)\right)\right)\\ -(1-d_{i})\log\left(1-G_{md}\left(G_{mf}\left(x_{{}^{i}}^{}\right)\right)\right) \end{array}$$

where $x_{i}^{}$ is the i-th sample of vibration signals, $G_{md}\left(\cdot \right)$ denotes the output of the marginal domain discriminator, and $d_{i}$ is the domain label. Notably, the source domain label and the target domain label are defined as 1 and 0, respectively.

The fused high-level conditional features from three spaces are linearly mapped to the output of the fault classifier. The linear mapping is used as an input of the conditional domain discriminator, which follows some interpretability for the conditional features containing potential category information. The loss function of the conditional domain discriminator can be formulated by:

(4) $$\begin{array}{l} L_{cd}=\left(G_{cd}\left(T_{⊗}\left(f_{i},{\hat{y}}_{i}\right)\right),d_{i}\right)=-d_{i}\log\left(G_{cd}\left(T_{⊗}\left(f_{i},{\hat{y}}_{i}\right)\right)\right)\\ -(1-d_{i})\log\left(1-G_{cd}\left(T_{⊗}\left(f_{i},{\hat{y}}_{i}\right)\right)\right) \end{array}$$

where $f_{i}$ is the high-level conditional features of the i-th sample, ${\hat{y}}_{i}$ is the output probability value of the Softmax function, $T_{⊗}\left(\cdot ,\cdot \right)$ denotes the linear mapping function, and $G_{cd}\left(\cdot \right)$ denotes the output result of the conditional domain discriminator.

The adaptive coefficient qualitatively and quantitatively combines the classification loss of the two domain discriminators above, which is calculated as follows:

(5) $$\mu =\frac{L_{cd}}{L_{cd}+L_{md}}$$

The smaller the conditional domain discriminator loss, $L_{cd}$, the more accurately the domain discriminator classifies the source or target domain. This dynamically adjusts the loss $L_{c}^{}$ according to the conditional distribution of the source domain and target domain. When the conditional distribution varies considerably, the weight for loss will be higher.

Table 2 shows the details of the marginal domain discriminator and the conditional domain discriminator. Layers F1, F2 and F3 are marginal domain discriminator layers. The conditional domain discriminator consists of layers F4, F5 and F6.

### 2.2. Training Process

The model aims to identify the fault type of the target domain sample by dynamically closing the marginal distribution and conditional distribution in two domains. The training process of the proposed DCMSDA model is detailed below.

Step 1: The labelled source and unlabeled target domain samples are treated as inputs, which are fed into the shared marginal feature extractor. MK-MMD loss for the marginal feature is calculated according to Equation (1).

Step 2: The extracted marginal features are further fed into the conditional feature extractors with three spaces, and LMMD loss for each space is calculated according to Equation (2).

Step 3: With the marginal features and the fused conditional features, the marginal and conditional domain discriminators, output the corresponding classification results, respectively. Then the binary loss for the two discriminators and the adaptive coefficient μ is calculated according to Equations (3)–(5).

Step 4: The fused high-level features are fed into the fault classifier and the source domain classification loss is expressed by:

(6) $$L_{y}=-\frac{1}{n}\left(\sum_{i=1}^{n}\sum_{j=1}^{C}1\left[y_{i}^{s}=j\right]\log\left(\frac{e^{\left(\theta_{j}{}^{T}\cdot x_{i}^{s}\right)}}{\sum_{l=1}^{C}e^{\left(\theta_{l}{}^{T}\cdot x_{i}^{s}\right)}}\right)\right)$$

where n denotes the number of samples in the source domain, C denotes the number of categories, log(·) denotes the output of Softmax function, 1[·] denotes the indicator function, and $\theta_{1},\theta_{2},\ldots ,\theta_{k}$ are learnable parameters in the fault classifier.

The proposed DCMSDA model has four optimization objectives: minimize MK-MMD loss, LMMD loss in three spaces, fault classification loss, and domain classification loss. Combined with these four loss functions, the overall optimization objective is as follows:

(7) $$L=L_{y}+\lambda \left[\mu L_{m}+\left(1-\mu \right)L_{c}\right]+(L_{md}+L_{cd})$$

where *λ* denotes the hyper-parameter to balance the whole loss, which is set to increase gradually from 0 to 1 as the iteration progresses.

Repeating the four steps of the above training process, the DCMSDA model is iteratively trained using the overall optimization objective. Through enough training, the feature extractors are able to extract domain-invariant fault features, thus allowing the shared fault classifier to accurately identify the fault type of target domain.

## 3. Experimental Verification

### 3.1. Experimental Dataset Description

**Figure 4:**
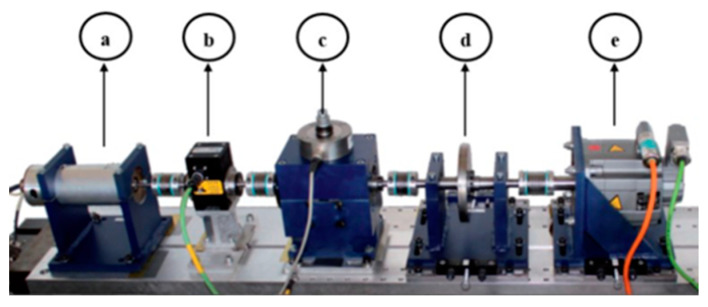
Experimental setup of PU: (**a**) electric motor; (**b**) torque measuring shaft; (**c**) rolling bearing test module; (**d**) flywheel; and (**e**) load motor.

To evaluate the effectiveness of the proposed bearing fault diagnosis method, the model, which is trained with labelled data, is expected to distinguish unlabeled data under different working conditions. The dataset provided by the University of Paderborn (PU) [20] is obtained from specially designed rolling bearings, including healthy state, inner raceway, and outer raceway faults. It contains 32 vibration signals, which can be spilit into healthy (6), artificially damaged (12), and real damaged (14), respectively, according to the accelerated lifetime, tested using 6203 grooved ball bearings [21]. Following [5], we only utilized the data on real damage. The modular test rig used to perform the accelerated life test is shown in Figure 4. The bearings used in the study were subjected to accelerated life by varying the rotational speed of the drive system, the radial force applied to the test bearings and the load torque on the driveline. In the test stage, a piezoelectric accelerometer was used to collect vibration signals from the bearing housing with a sampling frequency of 64 kHz.

The main operating parameters were the rotational speed, radial force, and load torque. Four diagnostic tasks are listed in Table 3. Thus, the domain adaptation task consisted of four different operating conditions. The proposed and compared methods were evaluated under 12 transfer tasks. For example, 0→1 denotes that the labelled data were taken as the source domain data, under the operation condition where task code 0 resides. On the contrary, the unlabeled data were utilized as target domain data.

In experiments, the bearings with actual damage were labelled at 0–12 for fault diagnosis tasks. Among the 13 faulty bearings, five failed in outer rings, five in inner rings, and the remaining failed in both inner and outer rings. Each fault category contained about 256,000 time-series sample points. With the segment length of 1024 data points, 250 samples per class were created, and each working condition contained about 250 × 13 samples. The four working conditions were composed of three working parameters, so the transfer difficulty was in line with the actual situation [22]. In addition, each fault category contained multiple fault parameters, such as main damage mode, damage degree and damage characteristics (the meaning of the specific content is explained in [20]). Therefore, the dataset for the experiments reflected potential variations of actual faults.

### 3.2. Training Details

The training parameter settings during the experiments are listed in Table 4. All experiments were conducted on a workstation as follows: CPU was Intel^®^ Core™ I7-6850K@ 3.60 ghz, GPU was GTX1080Ti, video memory was 11 GB. The operating system was Windows 10, and the deep learning framework was Pytorch1.10.

### 3.3. Compared Methods Description

To demonstrate that our method performed better than other models, seven models were selected for comparison under the same dataset.

#### 3.3.1. Domain Adaptation Based on Statistical Distance Metrics

According to [5], correlation alignment (CORAL) [23], MMD [24,25], MK-MMD, and Joint MMD (JMMD) [26] are often selected as statistical distance metrics. Here, we utilized the typical MK-MMD and JMMD as statistical distance metrics.

#### 3.3.2. Domain Adaptation Based on Adversarial Learning

We adopted two commonly used methods, including domain adversarial neural network (DANN) [27] and conditional domain adversarial network (CDAN) [28], as comparative models.

In addition, we compared Resnet, a deep learning method without domain adaptation; MRAN [29], an earlier proposed method with extracting features on multiple spaces; and DDAN [30], a representative method with dynamic distribution adaptation. For a fair comparison, the experimental configurations and the dataset were consistent across all models. The methods and their corresponding transfer modules are shown in Table 5.

### 3.4. Results and Analysis of Comparative Experiments

#### 3.4.1. Classification Accuracy

All methods under each transfer task were repeated 10 times with random initial parameters. Table 6 lists the average diagnostic accuracy of each method on the target domain. The average diagnosis accuracies of Resnet, DAN, JAN, DANN, CDAN, MRAN, DDAN, and the proposed DCMSDA were 50.31%, 69.44%, 72.38%, 73.51%, 73.79%, 71.25%, 71.34%, and 77.10%, respectively. In particular, for the simple transfer task of 2→0, the proposed model could still improve accuracy by 1.28%, compared with the CDAN model which already had an impressive accuracy. For the complex transfer task of 3→1, accuracies of the compared models were generally lower, while the proposed model obtained a high accuracy and outperformed the CDAN by 13%. All experimental results proved that our model could accurately classify faults in variable transfer tasks.

From Table 6 we can draw the following conclusions.

1. Comparison with Resnet:

Resnet does not use the domain adaption strategy, leading to significantly low accuracies under all tasks. The results indicate that for the scenario with variable working conditions, the classification performance of the model without domain adaptation may significantly reduce. However, our model overcomes this shortcoming.

2. Comparison with MRAN:

Although MRAN adopts the multi-space strategy, it only aligns the distribution of the high-level fault features over multiple spaces, ignoring the general information of vibration signals contained in the low-level features. In contrast, our model proposes two feature extraction modules, thereby providing more comprehensive information of faults.

3. Comparison with DDAN:

Although DDAN applies the dynamic adaptation strategy, it uses a linear classifier to calculate the adaptive coefficient, resulting in a poor fitting effect. Moreover, it only utilizes one feature extractor to extract features, which leads to singular fault features. In contrast, our model constructs non-linear domain discriminators to calculate the coefficient. Therefore, it is better matched to the actual working scenario.

4. Comparison with JAN:

JAN is a method based on joint distribution adaptation, and it assumes that marginal and conditional distribution adaptations are equally important. Therefore, when faced with complex transfer scenarios, it is impossible to quantify the relative importance of aligning two distributions, leading to a poor diagnosis. In contrast, benefiting from the adaptive coefficient, our model can automatically select the appropriate distribution alignment.

5. Comparison with CDAN:

CDAN achieves higher accuracy in the compared models. However, it only considers conditional distribution alignment. Moreover, this adversarial-based training approach usually faces the problem of slow convergence in model training. Thus, the diagnosis performance is inferior to our proposed method.

#### 3.4.2. Accuracy Curves

**Figure 5:**
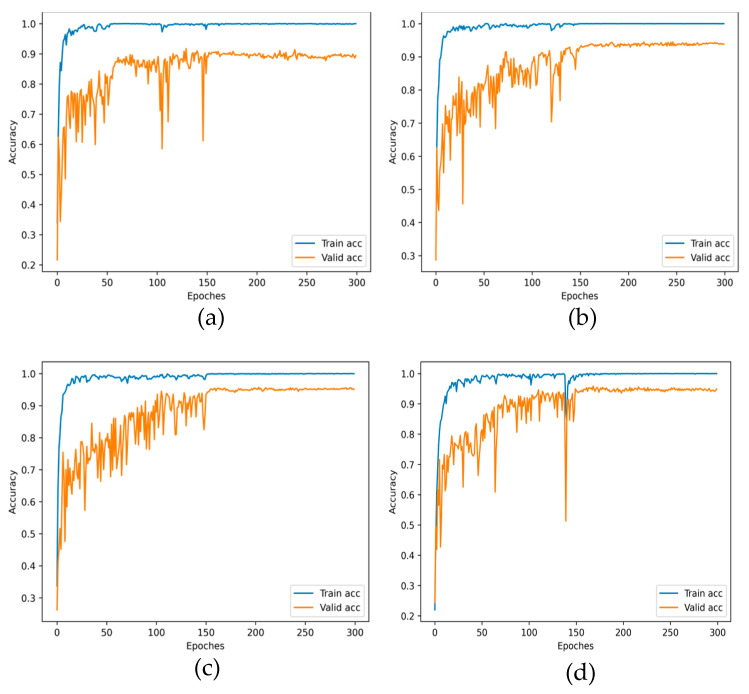
Training accuracy of source domain and test (Valid) accuracy of target domain for different models under the 2→0 transfer task: (**a**) DDAN; (**b**) JAN; (**c**) CDAN; and (**d**) proposed.

**Figure 6:**
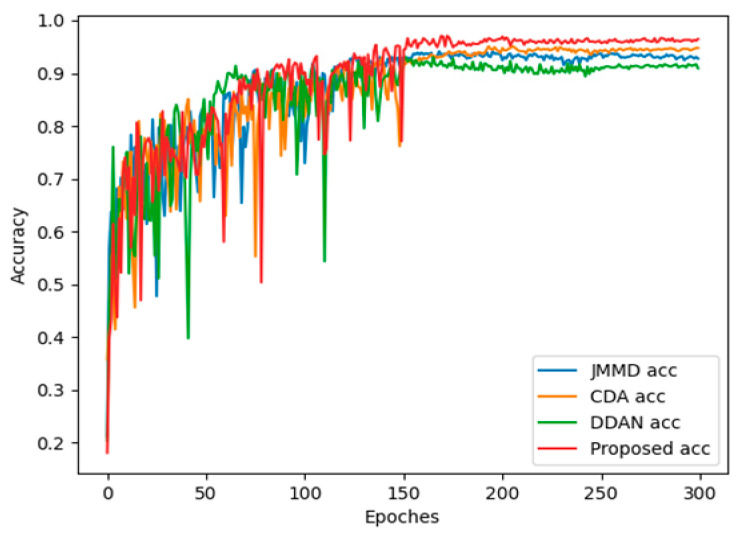
Test (Valid) accuracy of different models on target domain under the 2→0 transfer task.

To analyze the experimental results concisely, we chose the DDAN method with dynamic distribution adaptation, the JAN method with the best effect based on statistical distance metric, the CDAN method with the best effect based on adversarial learning, and our proposed model, as comparisons. Figure 5 shows the accuracy curves of DDAN, JAN, CDAN and the proposed DCMSDA model in the source domain training and the target domain test under the 2→0 transfer task, the optimal transfer task. There was a gap in accuracy between the source-domain training and target-domain test (Valid), which indicated that the distribution discrepancies between domains led to a lower generalization performance. Thanks to the proposed transfer modules, our model exhibited the smallest gap. This proved that the proposed model was easier to apply to actual working conditions. Additionally, in terms of the stability of the test (Valid) accuracy convergence, the proposed model significantly outperformed other methods. Figure 6 visually compares the test (Valid) accuracy curves for the four methods above. As can be seen, the accuracy of the proposed model was significantly higher than other methods.

#### 3.4.3. Feature Visualization

**Figure 7:**
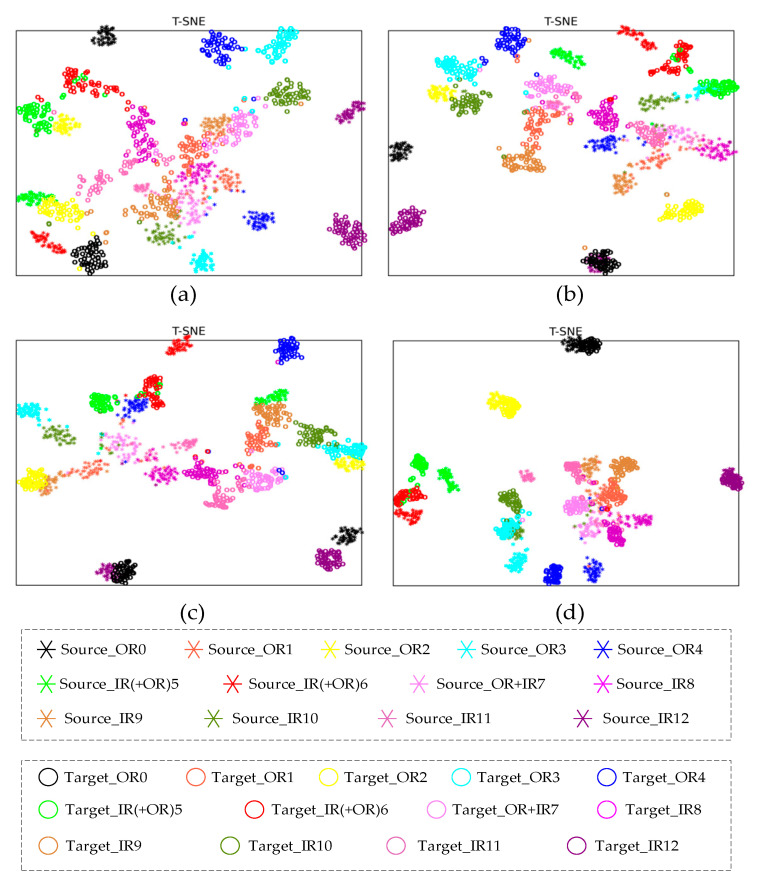
Feature visualization for different models under the 2→0 transfer task: (**a**) DDAN; (**b**) JAN; (**c**) CDAN; and (**d**) proposed.

To compare the domain alignment effects of different models, the fault classification results of DDAN, JAN, CDAN and the proposed DCMSDA model were visualized under the 2→0 transfer task using the t-distributed stochastic neighbor embedding (t-SNE) algorithm. As shown in Figure 7, two distinctive shapes indicate data from two domains, and diverse colors indicate varying fault categories. Some discussions of the feature representations are as follows:

1. The proposed DCMSDA model could obtain small intra-class distances and large inter-class distances, which suggests that our method has a strong fault classification capability. Specifically, as can be seen in Figure 7a, features in the DDAN method were somewhat jumbled with a poor gathering effect. Moreover, the category boundaries were not distinctly defined, which means that it was more difficult for the fault classifier to separate these features [31]. From Figure 7b,c, the JAN and CDAN methods incorrectly clustered the Source_IR12 fault and the Target_OR0 fault together, but the proposed method successfully separated them. From Figure 7d, the proposed method could achieve a better convergence effect of faults in the same category and obtain more obvious category boundaries;
2. The proposed DCMSDA model could extract representative domain-invariant features and exhibited excellent generalization performance because features of the source and target domains at the same fault category were closest. Specifically, as can be seen in Figure 7a–c, the three compared methods all closed the source domain and the target domain features of OR0, OR2 and IR12 faults unsuccessfully, but the proposed method closed them successfully, as shown in Figure 7d.

#### 3.4.4. Confusion Matrix

**Figure 8:**
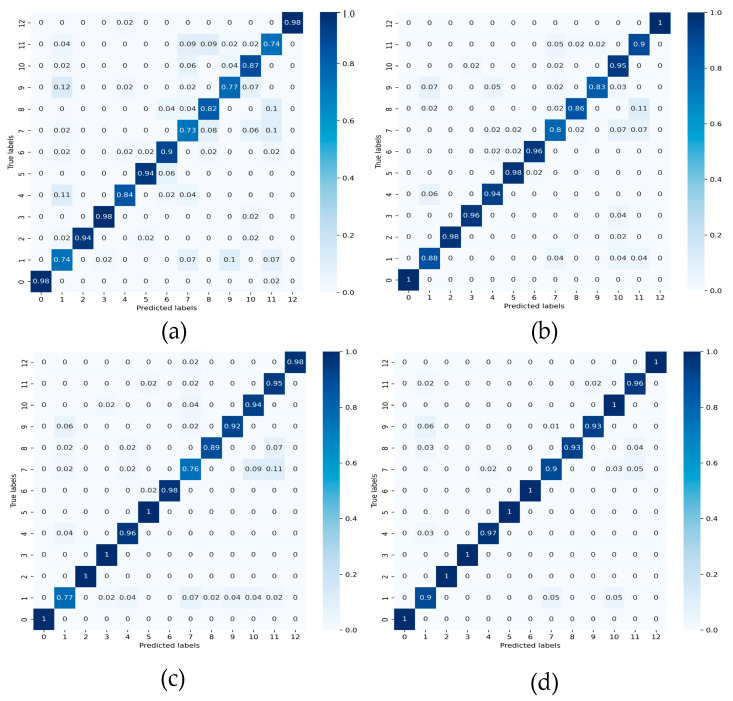
Confusion matrix for different models under the 2→0 task: (**a**) DDAN; (**b**) JAN; (**c**) CDAN; and (**d**) proposed.

The confusion matrices of DDAN, JAN, CDAN and the proposed DCMSDA model were compared under the 2→0 transfer task. Firstly, Figure 8a shows that DDAN had the lowest classification accuracy. The results in Figure 8b,c show that JAN had higher accuracy on certain label classification tasks, such as faults with label 1, and CDAN had higher accuracy on certain label classification tasks, such as label 9 and label 11. In contrast, the proposed model had higher accuracy than several other models on all label classification tasks, as shown in Figure 8d. Secondly, the results in Figure 8a–c show that when detecting the complex faults with label 7, diagnostic accuracies of the compared models were significantly lower, with only 73% for DDAN, 80% for JAN, and 76% for CDAN. In contrast, the proposed DCMSDA model could increase the accuracy to 90%, as shown in Figure 8d. All experiment results showed that the proposed model had excellent classification performance to identify variable types of faults.

#### 3.4.5. Receiver Operating Characteristics (RoC) Curves and Area under Curve (AuC) Values

**Figure 9:**
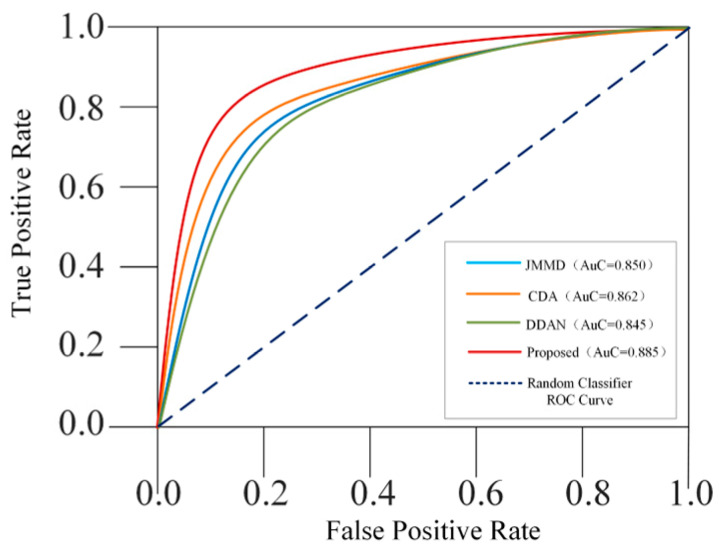
RoC curves and AuC values of classifiers for different models under the 2→0 task.

RoC curves of DDAN, JAN, CDAN and the proposed DCMSDA model were compared under the 2→0 transfer task, as shown in Figure 9. Here, we treated the Target_OR3 fault as the positive sample and the Target_OR4 fault as the negative sample. As can be seen, the proposed model had better classification performance because its RoC curve was closer to the coordinate point (0, 1), a point that only the perfect classifier would pass through. Moreover, we calculated the AuC value under each curve, as shown in the legend of Figure 9. The results showed that our model outperformed the other three models, as its AuC value was closer to 1.

### 3.5. Results and Analysis of Ablation Experiments

To verify the validity of the two feature extraction modules and the dynamic distribution adaptation module proposed in the model, we conducted experiments under the 3→1 transfer task with six cases. Specific cases and test accuracies are shown in Table 7.

The following conclusions can be drawn from Table 7.

1. Comparing cases 1 and 2 with the proposed model, two feature extraction modules focused on extracting informative features of vibration signals. The marginal feature extraction module could extract marginal features and obtain the general fault information. The multi-space conditional feature extraction module included convolution kernels of different depths and sizes, which could extract richer conditional features and obtain the information on fault categories, thereby guiding a more accurate result;
2. Comparing cases 3 and 4 with the proposed model, we adopted two different metrics to measure the distribution discrepancies, which contributed to exerting most of their respective strengths and guided the feature extraction modules to extract more diagnosis knowledge. MK-MMD focused on the global distribution and was suitable for aligning marginal features. LMMD was concerned with the relationship between two sub-domains within the same category, and was suitable for aligning conditional features;
3. Comparing case 5 with the proposed model, the adaptive coefficient dynamically measured the relative importance of marginal and conditional distribution alignments, thereby helping the model to adapt to complex cross-domain scenarios;
4. Comparing case 6 with the proposed model, the fault diagnosis model with domain adaptation aligned the distributions of domains, which significantly improved the robustness under the cross-condition diagnosis tasks;
5. Among them, experimental cases 3 and 5 with domain adaptation showed the largest reduction in accuracy compared with the proposed model. The results indicated that the strategy of adopting two metrics and the adaptive coefficient contributed the most to improving diagnostic accuracy.

## 4. Conclusions and Future Work

This paper presented a novel DCMSDA model based on domain adaptation for bearing fault diagnosis under cross-working conditions. It fully extracted domain-invariant features and achieved the alignment of the feature space. Firstly, the model considered the marginal distribution and conditional distribution discrepancies of the cross-domain vibration signals, as well as the applicability of different metrics. Therefore, two feature extraction modules were designed to extract the corresponding fault features separately, and two different metrics were adopted to align them. Then, an adaptive coefficient was employed to quantify the alignment proportion, so as to control the adaptation process. Through comparative experiments, the results demonstrated the superior performance of our method over current state-of-the-art methods, even in complex cross-domain scenarios. Furthermore, we verified the effectiveness of transfer modules in the proposed model through ablation experiments. The ablation results showed that the strategy of applying two metrics and the adaptive coefficient contributed the most to generalization performance.

Future work aims to find a more suitable multi-space integration strategy based on the variability of the fault features learned in each space, and to extend the proposed model to practical production applications, such as online fault diagnosis.

## Figures and Tables

**Table 1:** Architecture of the conditional feature extraction module.

Layer	Kernel Size	Kernel Number	Strides	Output Shape
CNN Block1	(1, 1)	64	1	(32, 64)
Average pooling	(7, 1)	64	1	(26, 64)
CNN Block2	(1, 1)	48	1	(32, 48)
CNN Block3	(5, 1)	64	1	(32, 64)
Average pooling	(7, 1)	64	1	(26, 64)
CNN Block4	(1, 1)	64	1	(32, 64)
CNN Block5	(3, 1)	96	1	(32, 96)
CNN Block6	(3, 1)	96	1	(32, 96)
Average pooling	(7, 1)	96	1	(26, 96)

**Table 2:** Architectures of the marginal and conditional domain discriminators.

Layer	Output Shape
F1: Fully connected marginal domain discriminator layer	(Batch_size, 1024)
F2: Fully connected marginal domain discriminator layer	(Batch_size, 1024)
F3: Fully connected marginal domain discriminator layer with one sigmoid	(Batch_size, 1)
F4: Fully connected conditional domain discriminator layer	(Batch_size, 1024)
F5: Fully connected conditional domain discriminator layer	(Batch_size, 1024)
F6: Fully connected conditional domain discriminator layer with one sigmoid	(Batch_size, 1)

**Table 3:** The diagnostic tasks of PU dataset.

Task Code	0	1	2	3
Load torque (Nm)	0.7	0.7	0.1	0.7
Radial force (N)	1000	1000	1000	400
Speed (rpm)	1500	900	1500	1500

**Table 4:** Training parameter settings during the experiments.

Parameter	Value	Parameter	Value
Epochs	300	Sample length	1024
Batch size	64	Marginal feature dimension	512
Weight decay	0.00001	Fused conditional feature dimension	256
Learning rate	0.001	-	-

**Table 5:** Methods and corresponding transfer modules used for fault diagnosis.

Method	Transfer Module	Adaptive Coefficient
Resnet	No transfer	No
DAN	MK-MMD	No
JAN	JMMD	No
DANN	Adversarial	No
CDAN	Condition-adversarial	No
MRAN	Multi-space	No
DDAN	MK-MMD and LMMD	Yes
Proposed	MK-MMD, LMMD and Multi-space	Yes

**Table 6:** Means of the test accuracies in different tasks with the PU dataset (%).

Transfer Task	Resnet	DAN	JAN	DANN	CDAN	MRAN	DDAN	Proposed
0→1	24.27	53.13	62.92	68.93	68.18	60.25	58.16	71.35
0→2	92.83	94.37	94.03	93.23	94.92	93.54	93.56	95.51
0→3	52.12	78.14	83.31	80.12	85.02	81.87	82.48	85.08
1→0	41.13	57.65	56.50	60.12	59.83	56.56	63.32	73.63
1→2	45.28	65.57	69.89	67.34	66.75	68.89	67.18	69.33
1→3	22.74	37.10	38.24	43.40	45.22	37.25	39.61	46.49
2→0	90.96	92.03	94.47	92.60	95.05	90.33	90.63	96.33
2→1	30.35	57.79	65.34	68.08	68.31	64.56	57.91	68.66
2→3	59.01	83.99	88.39	89.82	88.93	87.23	86.84	90.21
3→0	52.07	81.18	83.68	81.81	83.01	81.37	79.66	83.06
3→1	34.52	47.00	44.66	50.09	43.05	47.87	51.29	56.76
3→2	58.42	85.33	87.10	86.54	87.19	85.33	85.43	88.77
Average	50.31	69.44	72.38	73.51	73.79	71.25	71.34	77.10

**Table 7:** Cases and test accuracies of ablation experiments.

Case	Transfer Module	Adaptive Coefficient	Test Accuracy (%)
Case 1	MK-MMD, LMMD and marginal feature extraction module	Yes	55.21
Case 2	MK-MMD, LMMD and conditional feature extraction module	Yes	52.76
Case 3	MK-MMD and two feature extraction modules	Yes	39.26
Case 4	LMMD and two feature extraction modules	Yes	56.44
Case 5	MK-MMD, LMMD and two feature extraction modules	No	50.46
Case 6	Two feature extraction modules	No	30.54
Proposed	MK-MMD, LMMD and two feature extraction modules	Yes	57.98

## Data Availability

Publicly available datasets were analyzed in this study. This data can be found here: http://groups.uni-paderborn.de/kat/BearingDataCenter/ (accessed on 3 July 2022).
